# Investigating the Role of Response Codes in Masked Priming Lexical Decision Tasks: The Case of Repeated Presentations

**DOI:** 10.3390/brainsci13030452

**Published:** 2023-03-06

**Authors:** Maria Fernández-López, Ana Marcet, Manuel Perea

**Affiliations:** 1 ERI-Lectura, Universitat de València, 46010 Valencia, Spain; 2 Departamento de Metodología, Universitat de València, 46010 Valencia, Spain; 3 Departamento de Didáctica de la Lengua y la Literatura, Universitat de València, 46022 Valencia, Spain; 4 Centro de Investigación Nebrija en Cognición (CINC), Universidad Nebrija, 28015 Madrid, Spain

**Keywords:** masked priming, response congruency, lexical decision, repetition

## Abstract

The masked priming technique is considered a gold standard among experimental psychologists who specialize in the field of visual word recognition. Typically, this method entails a comparison between two or more critical conditions (e.g., the target word MOUSE being preceded by either the identity prime mouse or the unrelated prime fence). It is noteworthy that, unlike other masked priming tasks, prior experiments examining the properties of unrelated primes (e.g., their frequency as words [high or low] or their legality as nonwords [orthographically legal or illegal]) do not have an impact on the processing of the target item. However, two lexical decision studies reported faster responses to target words when the unrelated prime is a word rather than a nonword (i.e., a response congruency effect). One possible explanation for this discrepancy is a difference in methodology, as these two studies are the only ones to have used repeated presentation of stimuli, which could lead to the creation of an episodic memory trace that amplifies response congruency effects. To examine this hypothesis, we used a set of materials that did not show any congruency effect in a previous experiment with unique presentations, except that here we included repeated presentations. Results showed a response congruency effect, with participants responding faster to word targets when they were preceded by an unrelated word prime as opposed to an unrelated nonword prime. These findings suggest that the activation of response codes in masked priming is contingent upon the nature of cognitive resources required for processing the target stimuli.

## 1. Introduction

The most employed paradigm to unlock the initial phases of letter and word processing is the masked priming technique introduced by Forster and Davis [1,2,3,4]. In this technique, a target stimulus is briefly preceded (for 50 ms or less) by a masked prime, allowing researchers to examine the relationship between primes and targets (e.g., identity priming, orthographic priming, translation priming, semantic priming, etc.). For instance, when investigating identity priming, the usual comparison would be between pairs such as table-TABLE (identity prime) vs. phone-TABLE (unrelated prime) [1]. This technique has been utilized in various measures, including response time collection, electrophysiology, and brain activity (neuroimaging and magnetoencephalography) [5,6,7,8].

Masked priming experiments have given us valuable insights into how the brain processes written language. These findings have expanded our knowledge, not just in mature word recognition systems (see [4] for review), but also in the developing brain (see [9,10,11,12] for studies with neurotypical developing readers; see [13] for dyslexic readers). Notably, these findings have had crucial implications for models of word recognition (e.g., the dual-route cascaded model, the spatial coding model, the multiple read-out model, the Bayesian Reader model, and the dual-route model of orthographic development) [14,15,16,17,18].

Masked priming can be used for various tasks such as lexical decision, same–different tasks, picture naming, semantic categorization, alphabetic decision, stem completion, and letter detection, among others. However, the present study focuses on the most commonly used task in visual word recognition research, the lexical decision task. This task involves determining whether a letter string is a word or not. Our focus in the present study is on a methodological issue with significant implications at theoretical and methodological levels: whether the characteristics of the unrelated prime (e.g., lexicality [word vs. pseudoword], word frequency, and orthographic legality) influence target processing. Response congruency effects refer to the difference in response times between trials where the prime elicits the same response as the target (congruent trials) versus trials where the prime elicits a different response than the target (incongruent trials). Importantly, examining response congruency effects in masked priming lexical decision allows us to scrutinize the state of the lexicon once the prime is processed up to an early stage [19]. Thus, studying this effect helps to deeply elucidate the processes underlying the first moments of lexical access. Indeed, interactive activation models, e.g., [14,16] have been used to simulate response patterns in lexical decision tasks. These simulations revealed that response times to a target word are generally faster when preceded by a word prime, particularly a high-frequency word, compared to a nonword prime (see [19,20,21] for discussion).

Previous masked priming studies have shown that response congruency affects tasks other than lexical decision [22,23]. For example, in a numerical categorization task (“is the number higher than 5?”), Naccache and Dehaene [22] found that participants gave faster responses when the masked prime and target were in the same category (e.g., 7 [prime]–8 [target], both >5), as opposed to different categories (e.g., 2 [prime, <5]–9 [target, >5]). Similarly, in a masked semantic categorization task, Forster [23] found that, for narrow categories (“is the item a type of dog?”), participants showed a response congruency effect (e.g., responses were faster to answer-WINDOW than collie-WINDOW).

Sereno [24] was the first to investigate the potential impact of the lexical status of the unrelated condition on masked priming effects in lexical decision. To accomplish this, the author presented target words such as BEAST with an identity prime (beast), an unrelated word prime (e.g., enter), and an unrelated nonword prime (pasil). Similarly, for target nonwords such as PLAVE, they presented an identity prime (plave), an unrelated word prime (month), and an unrelated non-word prime (blask). Results indicated that lexical decision times were faster in the identity priming condition than in the unrelated conditions, which did not differ from each other (enter-BEAST ≈ pasil-BEAST; month-PLAVE ≈ blask-PLAVE). Along the same lines, Perea et al. examined the role of the lexical status (word vs. nonword) and the frequency (high-frequency word vs. low-frequency word) of the unrelated primes for both word and nonword targets. They found a similar advantage of the identity priming condition over the other conditions, which did not differ from each other ([25], see also [17,20], for similar null findings).

In a recent series of experiments, Fernández-López et al. [21] re-examined this issue, including high-frequency unrelated word primes, low-frequency unrelated word primes, orthographically legal pseudoword primes, and consonant string primes. When the nonword targets in lexical decision were matched with the target words in the critical parameters (Experiment 1), the authors did not find any differences among the unrelated conditions. Notably, when all the nonword targets were orthographically and phonologically illegal (e.g., BEFRM, Experiment 2), the legality of the unrelated nonword primes played a role: consonant string unrelated primes led to faster responses to nonword targets, but slower to word targets. The authors attributed these differences to the fact that, as all nonword targets were illegal, participants made an orthographic legality decision in which the legality of the primes helped the word/nonword decision. Indeed, for the word targets, there were no effects of word frequency, thus suggesting that the task did not involve the typical processes underlying lexical decision. Finally, in Experiment 3, the nonword target had no lexical neighbors but was orthographically and phonologically legal (e.g., the target word ERTOL). Fernández-López et al. [21] found no differences among the unrelated priming conditions in this third experiment.

Thus, the above-cited lexical decision experiments have revealed an absence of an effect due to the frequency and lexical status of the unrelated primes, at least when using the typical setup (i.e., orthographically legal nonword targets). However, two published experiments have reported differences due to the lexical status of the unrelated primes [26,27]. Critically, there is one methodological difference with the above experiments: these two studies involved a repeated presentation of the stimuli, whereas in the other experiments, each item was presented only once. Jacobs et al. [26] used a variant of the masked priming technique that involved the repetition of the stimuli across four luminance conditions and included two unrelated priming conditions (an unrelated word prime and an unrelated nonword prime). For word targets, they found faster responses when the unrelated prime was a word rather than a nonword (house-TABLE < geuze-TABLE). In contrast, the opposite trend occurred for nonword targets (geuze-ABINO < house-ABINO). Jacobs et al. [26] interpreted this dissociation in terms of the activation of response codes (i.e., “yes” for words vs. “no” for nonwords). When primes and targets share the response code, there would be less inhibition from the unrelated primes than when they do not share the response code. In addition, Perea et al. [27] conducted two experiments, including unrelated word primes and nonword primes in a masked identity priming task. In the first experiment, the stimuli were presented only once, obtaining no effect of the lexical status of the unrelated prime. Notably, in the second experiment, the set of items involved four repetitions of each target. They found, for word targets, faster lexical decision times when the prime was an unrelated word than an unrelated nonword (house-TABLE < geuze-TABLE). However, for nonword targets, they found no effect of the lexical status of the unrelated primes (geuze-ABINO ≈ house-ABINO). Thus, at least for word targets and repeated presentations, there seems to be some evidence of a congruency effect in lexical decision when the response codes are the same for primes and targets, thereby resembling the effects reported in other masked priming tasks (e.g., numerical categorization, semantic categorization) [22,23].

To reconcile the disparities between the lexical decision experiments that featured a single presentation of the target stimuli and those with repeated exposures, it could be argued that the repetition of stimuli may activate distinct processing mechanisms during word recognition. For instance, in Logan’s [28] model, repeated presentations induce the activation of episodic traces that include response codes. Notably, these episodic traces would be absent when the items are presented only once. However, it is difficult to reach firm conclusions as the above experiments with repeated presentations did not directly test the same materials in a scenario with unique presentations.

The present lexical decision experiment was designed to examine whether the lexical status and word frequency of the unrelated masked primes play a role when there are repeated presentations. To allow for a direct comparison with the standard scenario (i.e., non-repeated targets), we used a set of materials that did not show any signs of differences among the unrelated priming conditions with a unique presentation (Fernández-López et al.’s Experiment 3) [21]. We selected a subset of the target stimuli to keep the same number of trials as in Fernández-López et al.’s experiment. Notably, this design allowed us to have four unrelated priming conditions (high-frequency word, low-frequency word, pseudoword, and consonant string) and test three planned contrasts: the lexical status of the unrelated prime (high- and low-frequency words vs. pseudowords and consonant strings), word frequency (high- vs. low-frequency words), and the legality of the nonword (legal [pseudowords] vs. illegal [consonant strings]).

If shared response codes between primes and targets play a role in masked priming when there are repeated stimuli presentations, we expect to find a dissociative congruency effect for word and nonword targets. In this case, unrelated word primes would facilitate responses to word targets relative to unrelated nonword primes, whereas the opposite trend would occur for nonword targets. In addition, the word frequency and legality of the primes might also shape the above, with the effects being stronger for high-frequency words than low-frequency words, and for pseudoword primes than for consonant string primes—the opposite effects would be predicted for nonword targets. In this scenario, current models of visual word recognition should consider that episodic traces from earlier presentations may modulate orthographic processing. Alternatively, if the response codes are not activated in masked priming lexical decision, even in repeated exposures, one would expect similar lexical decision times and error rates across the four unrelated priming conditions. This finding would suggest that congruency effects in masked priming lexical decision may be specific to specific situations.

## 2. Materials and Methods

### 2.1. Participants

The sample consisted of twenty-four first-year undergraduate students from the University of València. This sample size was identical to that of the experiments conducted by Fernández-López et al. [21]. It allowed us to obtain 1440 observations (24 ∗ 40 = 1440) in each priming condition, which is in line with Brysbaert and Stevens’ [29] recommendations for masked priming experiments. All participants were native Spanish speakers with no prior reading difficulties. The experiment was approved by the Ethics Research Committee of the University of València, and the participants provided their informed consent before the study commenced.

### 2.2. Materials

We employed a subset of the materials from Fernández-López et al.’s [21] Experiment 3, all with five letters. The target stimuli were 60 Spanish words (e.g., ABEJA [bee], mean Zipf frequency = 4.14; range: 3.74–4.61), and 60 orthographically legal pseudowords with no lexical neighbors (e.g., ERTOL) [30]. There were four conditions: each target item, consistently displayed in uppercase, was preceded by an unrelated lowercase prime that could be (1) a high-frequency word (e.g., fuego [fire], mean Zipf frequency = 5.23, range: 4.74–5.95); (2) a low-frequency word (e.g., secta [cult], mean Zipf frequency = 3.25, range: 2.17–3.68); (3) an orthographically legal pseudoword (e.g., mucor); or (4) a consonant string (e.g., zglnc). In a Latin Square design, each target item underwent rotation across the four conditions in 120 trials. Participants were presented with the four lists—the list order was random. Thus, each participant received a total of 480 trials, split evenly between word trials (240) and nonword trials (240), with 120 trials in each priming condition.

### 2.3. Procedure

The experiment was conducted in a laboratory setting, with groups of three to four participants. The programming of the experiment was performed using DMDX [31]. Participants were instructed to determine whether a presented letter string constituted a word or not as quickly and accurately as possible, indicated by pressing the “yes” or “no” key, respectively. On each trial, the computer screen first displayed a series of hash marks (#####) for 500 milliseconds, followed by a lowercase prime for 50 milliseconds, and then, the target stimulus in uppercase. The target stimulus remained on the screen until the participant responded or until a 2 s deadline had passed. In this latter case, the response was categorized as an error. The stimuli were presented in black (Courier New 16 pt) on a white background. Participants were not informed about the item repetitions across blocks. The session lasted approximately 25 min and included a practice phase consisting of 16 trials before the experimental phase and a break every 120 trials during the experimental phase.

### 2.4. Analyses

We created separate linear mixed-effects models for the correct response times (RTs), where the fixed factors were lexicality (word [−0.5] and nonword [0.5]) and type of unrelated prime (high-frequency word, low-frequency word, pseudoword, consonant string), using the lme4 package in R [32,33]. To obtain the *p* values for the effects, we employed Satterthwaite’s method of the lmerTest package [34]. We used a −1000/RT transformation to address the positive skew of response time distributions. We also followed the same criteria as in the Fernández-López et al. experiments to test three planned orthogonal contrasts of the type of prime: (1) the effect of the lexical status of the unrelated prime: word prime (combining high- and low-frequency words) vs. nonword prime (combining pseudowords and consonant strings); (2) the effect of the frequency of the unrelated word prime: high-frequency word vs. low-frequency word; and (3) the effect of the legality of the unrelated nonword prime: pseudoword vs. consonant string. We employed the maximal random-effect structure in subjects’ and items’ intercepts and slopes that converged successfully.

In addition, we also sought to confirm our inferential analyses through a descriptive quantile-based approach. To accomplish this, we examined the impact of each priming condition across each decile (i.e., Quantile 0.1, Quantile 0.2, …, Quantile 0.9).

## 3. Results

Response times faster than 250 ms (2 trials overall; less than 0.02% of trials) and incorrect responses (2.81% for words; 2.97% for nonwords) were omitted from the response time analyses. The mean correct lexical decision times and percentage accuracy in each of the conditions of prime type (high-frequency word, low-frequency word, pseudoword, consonant string) and lexicality (word, pseudoword) are presented in Table 1. As accuracy was at very high levels in all conditions, further analysis of accuracy was not deemed necessary.

### 3.1. Effect of Lexical Status of the Unrelated Prime (Words vs. Nonwords)

While the overall effect of the lexical status of the prime did not approach significance (*b* = −0.013, *SE* = 0.017, *t* = 0.80, *p* = 0.43), we found an interaction of this factor with lexicality (*b* = −0.059, *SE* = 0.023, *t* = 2.53, *p* = 0.01). This interaction revealed that for word targets, response times were faster when preceded by an unrelated word prime than when preceded by an unrelated nonword prime (*b* = −0.047, *SE* = 0.018, *t* = 2.56, *p* = 0.01). In contrast, nonword targets did not reveal an effect due to the lexical status of the primes (*b* = 0.016, *t* = 0.92, *p* = 0.36).

### 3.2. Effect of Word Frequency of the Unrelated Word Prime (High- vs. Low-Frequency Words)

There were no signs of an effect of the frequency of the primes (*t* = 0.71, *p* = 0.48) or an interaction with lexicality (*t* = −1.11, *p* = 0.27).

### 3.3. Effect of Legality of the Nonword Unrelated Prime (Pseudowords vs. Consonant Strings) 

Response times were slightly higher when the prime was composed of consonant strings than when the prime was a pseudoword, but the difference was not significant (*b* = −0.019, *SE* = 0.012, *t* = 1.60, *p* = 0.11). This pattern occurred regardless of the lexicality of the target (interaction: *t* = 0.20, *p* = 0.84).

### 3.4. Descriptive Quantile-Based Approach 

**Figure 1:**
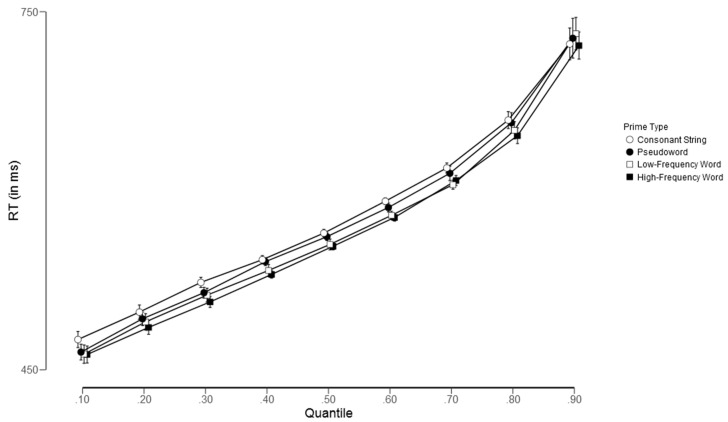
Group reaction time distributions for the four experimental conditions across quantiles (0.1, 0.2, …, 0.9) for word targets.

**Figure 2:**
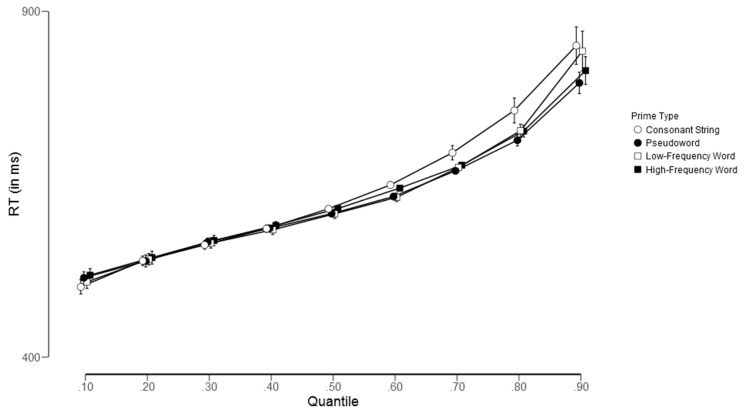
Group reaction time distributions for the four experimental conditions across quantiles (0.1, 0.2, …, 0.9) for nonword targets.

As shown in Figure 1 and Figure 2, the effect of prime type on word targets was highly consistent across quantiles, indicating that more word-like primes produced faster response times compared to less word-like primes, thereby reflecting the influence of lexical status (see Figure 1). Notably, the pattern for nonword targets was different: all types of primes exhibited similar behavior along the RT distributions, but there was a slight disadvantage for consonant string primes in higher quantiles (see Figure 2).

## 4. Discussion

The present lexical decision experiment aimed to address a puzzling discrepancy in masked priming findings. The vast majority of experiments consistently showed a lack of a congruency effect when using unrelated primes, regardless of their frequency or lexicality, as reviewed in [21]. However, two studies reported a congruency effect for word targets [26,27]. One key methodological difference between these studies is that the targets in the studies by Jacobs et al. [26] and Perea et al. [27] were repeatedly presented, which may have influenced the memory traces during lexicon access [28].

To resolve this discrepancy, we used the materials of Fernández-López et al. [21] in a scenario where the target stimuli were presented several times. Our findings revealed that the reaction times for word targets in the lexical decision task were faster when preceded by a word prime than when preceded by a nonword prime, yielding a response congruency effect. These results replicated the findings of Jacobs et al. [26] and Perea et al. [27]. Notably, as indicated above, our materials did not exhibit a congruency effect in the absence of repetition [21]. Additionally, as in the Perea et al. [27] experiment, we found no evidence of a congruency effect for nonword targets.

Thus, it can be concluded that response congruency effects can be observed in lexical decision experiments utilizing masked priming. However, these effects are limited to scenarios in which the items are repeated several times. While response congruency effects have been found to be consistent for word targets (as demonstrated in the current experiment, Jacobs et al. [26], and Perea et al. [27]), this consistency is less evident for nonword targets (as shown in Figure 2). It should be noted that masked priming effects for nonword targets are typically more variable and less robust than those for word targets, likely because a “no” response can be based on multiple sources of information, such as lexical search or a temporal deadline [19]. At a theoretical level, as previously indicated in the Introduction, interactive activation models have been shown to effectively predict response patterns for word targets (see [19,20]), while simulation studies of the Bayesian Reader model [17] have predicted a null congruency effect for word targets (see [17,19]). These findings suggest that, for repeated stimulus presentations, interactive activation models offer better predictions of response congruency effects than the Bayesian Reader model. Future research could benefit from more nuanced integration of lexical access processes and parameters relating to the flexibility of lexical representations in the context of stimulus repetition.

The presence of response congruency effects in masked priming, as observed through the lexical decision task with several repetitions, is comparable to those reported through other tasks. As reviewed in the Introduction, response congruency effects have been observed in numerical categorization tasks that employed multiple repetitions of the same target stimuli [22]. These findings suggest that the cognitive system may use prime information, in the form of a response code, under certain conditions that include episodic traces. Nonetheless, it is noteworthy that repetition is not the sole factor influencing response congruency effects in masked priming. Response congruency effects in masked priming can also occur through semantic categorization tasks without repetitions, though only when the category is narrow [23]. Furthermore, response congruency effects in lexical decision can be elicited when the nonword targets are orthographically and phonologically illegal (e.g., TFCRO). Note that this scenario transforms the lexical decision task into an “orthographic legality” task [21].

Taken together, our findings suggest that future studies emphasize the importance of designing an appropriate methodological scenario, which entails selecting a proper set of stimuli, establishing a suitable baseline, and defining the task to ensure the accurate interpretation of results in masked priming experiments. We acknowledge that the subtleties of the congruence effect for word targets, specifically the marginal differences observed in response times when the prime was a high-frequency word compared to a low-frequency word, as well as the marginal differences observed in response times when the prime was a pseudoword compared to a consonant string, could potentially be captured with a considerably larger sample size. However, even if these minute differences were to persist in this scenario, the core implications of our study remain unaltered, namely, that a set of stimuli that do not elicit congruency effects in masked priming under typical conditions is capable of producing congruency effects through repeated presentation. Another issue worth studying in future research is whether the response congruency effect with repeated presentations is modulated by the characteristics of the previous prime-target pairing (e.g., whether an unrelated word or nonword prime in lexical decision tasks, or whether a prime higher than 5 or lower than 5 in numerosity categorization tasks).

## 5. Conclusions

In sum, this theoretical and methodological paper has demonstrated that the activation of response codes in masked priming is likely contingent upon the cognitive resources and type of processing required for the target stimuli. Scenarios that are easier to process may result in the “decision” made on the prime features affecting the processing of the subsequent target item, even when there is no apparent relation between them. To test this hypothesis in future research, researchers could incorporate various levels of cognitive load in a masked priming paradigm (e.g., [35]). 

## Figures and Tables

**Table 1:** Mean correct lexical decision times (in ms) and % accuracy (in brackets) for words and pseudowords in the experiment.

	Prime Type			
	High-Freq Word	Low-Freq Word	Pseudoword	Consonant String
Word	579 (97.9)	585 (96.7)	586 (96.9)	591 (97.2)
Pseudoword	650 (97.1)	647 (96.9)	642 (97.1)	659 (97.1)

## Data Availability

The stimuli, data, scripts, and results are available at the following link: https://osf.io/2t4da/?view_only=517db5274438448393a9e8dd781adf0c (accessed on 2 March 2023).
